# Optimization of decellularization methods using human small intestinal submucosa for scaffold generation in regenerative medicine

**DOI:** 10.1111/iep.12492

**Published:** 2023-08-25

**Authors:** Shumei Mineta, Shunji Endo, Tomio Ueno

**Affiliations:** ^1^ Department of Digestive Surgery Kawasaki Medical School Kurashiki Japan

**Keywords:** decellularization, extracellular matrix, small intestinal submucosa

## Abstract

Porcine small intestinal submucosa, despite its successful use as a scaffold in regenerative medicine, has innate biomechanical heterogeneity. In this study, we hypothesized that human small intestinal submucosa could be a viable alternative bio‐scaffold. For the first time, we characterize submucosal extraction from human small intestine and examine appropriate decellularization methods. In total, 16 human small intestinal submucosal samples were obtained and decellularized using three reported methods of porcine decellularization: Abraham, Badylak, and Luo. For each method, four specimens were decellularized. The remaining four specimens were designated as non‐decellularized. We measured the amount of residual DNA and growth factors in decellularized human intestinal samples. Additionally, decellularized human small intestinal submucosa was co‐cultured with mouse bone marrow‐derived mesenchymal stem cells to examine mesenchymal stem cell survival and proliferation. The reference value for the amount of residual DNA deemed appropriate in decellularized tissue was established as 50 ng/mg of extracellular matrix dry weight or less. Abraham's method most successfully met this criterion. Measurement of residual growth factors revealed low levels observed in samples decellularized using the Abraham and Badylak methods. Co‐culture of each small intestinal submucosal sample with mouse bone marrow‐derived mesenchymal stem cells confirmed viable cell survival and proliferation in samples derived using protocols by Abraham and Badylak. Abraham's method most successfully met the criteria for efficient tissue decellularization and cell viability and proliferation. Thus, we consider this method most suitable for decellularization of human small intestinal submucosa.

## 1 INTRODUCTION

Regenerative medicine often involves xenogeneic scaffold generation for the purposes of implantation to repair non‐functional tissues or organs. Three factors are crucial for the successful regeneration of tissues and organs: appropriate “cells”, appropriate “scaffold materials” and appropriate “growth factors”. Induced pluripotent stem (iPS) cells have emerged as ideal “cells” in regenerative medicine, but their regenerative capacity is ultimately reliant on appropriate “scaffold materials” and “growth factors^”^.^1^ The submucosa derived from the porcine small intestine (SIS) includes the mucous membrane and serosal muscle layer, which consists of supramolecular organized extracellular matrix and growth factors. This SIS is directly involved in the control of cell retention, proliferation, and differentiation, and is widely used as a scaffold for regeneration of human tissues such as arteries,^2^ bladder,^3^ intestine,^4^, ^5^ and tendons.^6^

Because porcine SIS is heterogeneous, decellularization is necessary for human regenerative medicine. In theory, an ideal method for decellularization removes cellular components yet preserves growth factors without damaging the extracellular matrix. Various methods have been reported.^7^, ^8^, ^9^ However, even if decellularized porcine SIS is used, concerns about its use in human regenerative medicine have been raised^10^ because these xenografts may elicit immune responses either against cellular debris or intact heterologous proteins.^11^ In addition, the “gal epitope” expressed in the cell membranes of all mammals, except humans and primates, is thought to be the cause of rejection.^11^

We theorized that generation of human‐derived scaffolds would be an ideal alternative to porcine tissue. However, it was unclear which decellularization method would be most appropriate for use with human SIS. This study is the first to evaluate whether porcine decellularization methods may be successfully applied to human tissue.

## 2 MATERIALS AND METHODS

### 2.1 Collection of human small intestine

Human small intestinal tissue collection was performed in accordance with ethical approval from the Kawasaki Medical School Ethics Committee (approval number: 5219‐00). Written informed consent was obtained from all recruited patients undergoing surgery involving partial resection of normal small bowel as part of standard surgical treatment (including pancreatoduodenectomy, ileocecal resection, and small bowel tumour resection). We collected a total of 16 normal small bowel specimens.

### 2.2 Fabrication and decellularization of human SIS

We decellularized human SIS using three established protocols: Abraham's method (A‐SIS),^7^ Badylak's method (B‐SIS),^8^ and Luo's method (L‐SIS).^9^ SIS without decellularization was labelled as “N‐SIS”. Each decellularization method is described below.

### 2.3 A‐SIS

The serosa and muscle layer of the small intestine was isolated and incubated in a solution of 100 mM ethylenediaminetetraacetic acid (EDTA)/10 mM of sodium hydroxide (NaOH) (pH 12) for 16 h. Then, incubation in 1 M of hydrochloric acid (HCL)/1 M sodium chloride (NaCl) (pH 1) was carried out for 8 h followed by another incubation in 1 M of NaCl and 10 mM of phosphate‐buffered saline (PBS) (pH 7.4) for 16 h. The sample was then incubated in 10 mM PBS (pH 7.4) for 2 h. Finally, the SIS was washed in sterile water (pH 7.0) for 2 h.

### 2.4 B‐SIS

The small intestine sample was snap‐frozen at −80°C. After thawing, the serosa and muscle layer of the small intestine was isolated and shaken (250 rpm) for 2 h in a 0.1% peracetic acid/4% ethanol solution (100 mL per 10 g wet weight). The SIS was washed twice (15 min) with buffered saline followed by a further two washes (15 min) in deionized water.

### 2.5 L‐SIS

After the serosa and muscle layer of the small intestine were isolated, the sample was washed with PBS. The SIS was soaked in a solution containing methanol and chloroform (1:1, V/V) for 12 h and rinsed with deionized water. This was followed by incubation in 0.05% trypsin/0.05% EDTA for 12 h at 37°C. The sample was rinsed with saline and then continuously shaken in 0.5% sodium dodecyl sulphate in 0.9% NaCl for 4 h. The detergent was then removed by rinsing thoroughly with saline. Finally, the SIS was soaked in 0.1% peracetic acid and 20% ethanol for 30 min and rinsed with saline.

### 2.6 Observation with an optical microscope

Each tissue section of decellularized human SIS was confirmed by haematoxylin–eosin (HE) staining. Specifically, SIS was fixed with formalin, stained with HE, observed with an optical microscope, and photographed with a camera.

### 2.7 Measurement of residual DNA content of decellularized human SIS

We extracted the DNA of each decellularized SIS and measured the amount of residual DNA. Specifically, we freeze‐dried decellularized SIS and extracted DNA using the QIAamp DNA Mini Kit™ (QIAGEN, Venlo, Netherlands). We quantified the extract with Quant‐iT™ PicoGreen™ dsDNA Assay Kits and dsDNA Reagents (Thermos Fisher Scientific, Waltham, MA, USA) to evaluate the amount of residual DNA.^12^

### 2.8 Measurement of growth factor content in decellularized human SIS

Reports suggest that three main growth factors are found in SIS: fibroblast growth factor (FGF), vascular endothelial growth factor (VEGF) and transforming growth factor‐β (TGF‐β).^13^, ^14^, ^15^ We measured the content of these growth factors in N‐SIS and each decellularized SIS. All samples were lyophilized and the measurement protocol was undertaken according to that reported by Chun et al.^14^ The growth factor extraction buffer was altered specifically for each growth factor measured^14^; WSE‐7420 EzRIPA Lysis kit™ (ATTO, Tokyo, Japan) was used for extraction of FGF while the extraction buffer outlined by Chun et al.^16^ was used for extraction of VEGF and TGF‐β.

Extraction buffer containing protease inhibitors was added to each lyophilized SIS sample. The sample was left at 4°C on gentle agitation for 1–3 days. The solution was centrifuged at 12,000 × *g* for 30 min at 4°C. The supernatant was harvested and dialyzed at 4°C for 2 days using a semi‐permeable membrane with a 3500‐Dalton protein MW cut‐off (MWCO) (Slide‐A‐Lyzer™, Thermos Fisher Scientific). The dialysate was centrifuged at 12,000 × *g* for 30 min at 4°C. The supernatant was collected and each growth factor was quantified by enzyme‐linked immunosorbent assay (ELISA): FGF using ELISA MAX™ Deluxe Set Human FGF‐basic (BioLegend, San Diego, CA, USA), VEGF using VEGF‐A Human ELISA Kit (Thermo Fisher Scientific), and TGF‐β using Human TGF‐beta 1 DuoSet ELISA (R&D Systems, Minneapolis, MN, USA).

### 2.9 Evaluation of cell proliferation and survival by co‐culture of decellularized SIS and mouse bone marrow‐derived mesenchymal stem cells (BMSCs)

Murine BMSCs were co‐cultured with all three types of decellularized human SIS and cell proliferation and survival were quantified according to the protocol outlined by Yanhui et al.^12^

A 5 × 5 mm square sheet of each decellularized SIS was placed in a single well of a 96‐well plate and co‐cultured with BMSCs. The SIS was removed after 0, 3, and 7 days and the BMSCs engrafted to the SIS stained with Cellstain® Calcein‐AM solution (Fujifilm, Tokyo, Japan), which is a live cell viability stain. The sample was visualized using a fluorescence microscope. WST‐1 Assay Reagent (Premix WST‐1 Cell Proliferation Assay System™, Takara Bio, Kusatsu, Japan) was added to the remaining medium without SIS and the absorbance was measured to quantify the proliferation rate of BMSCs.

### 2.10 Data analysis

We performed statistical analysis using JMP®9 (sas Institute Inc. Cary, NC, USA) with numerical data presented as the median (lower range value – upper range value). The non‐parametric Mann–Whitney *U* test was used for comparing differences between each independent group and a *p* value <.05 was considered to be significant.

## 3 RESULTS

### 3.1 Observation of residual cells with an optical microscope

**FIGURE 1:**
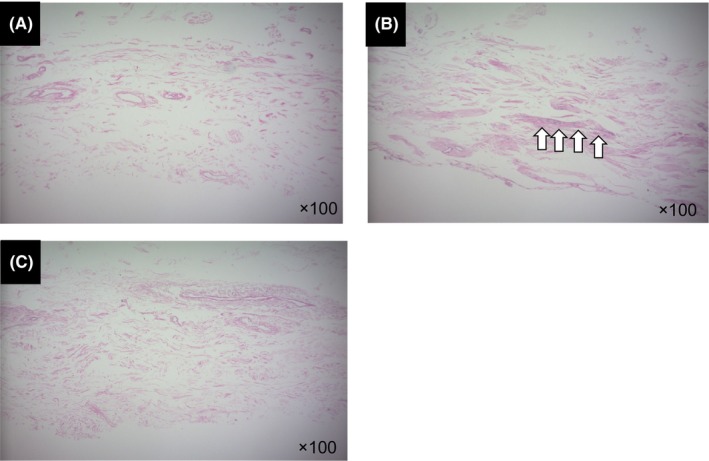
Representative histological images of decellularized human small intestinal submucosal (SIS) samples by haematoxylin–eosin staining. (A–C) demonstrate cross‐sectional images of Abraham's method (A‐SIS), Badylak's method (B‐SIS), and Luo's method (L‐SIS), respectively. Residual cells were observed in B‐SIS whereas no cells were observed in A‐SIS and L‐SIS. Remaining cells are illustrated by arrows (⇧).

Residual cells were observed in samples decellularized using the B‐SIS method whereas no cells were observed in samples derived using both the A‐SIS and L‐SIS methods (Figure 1).

### 3.2 Amount of residual DNA in decellularized human SIS

**FIGURE 2:**
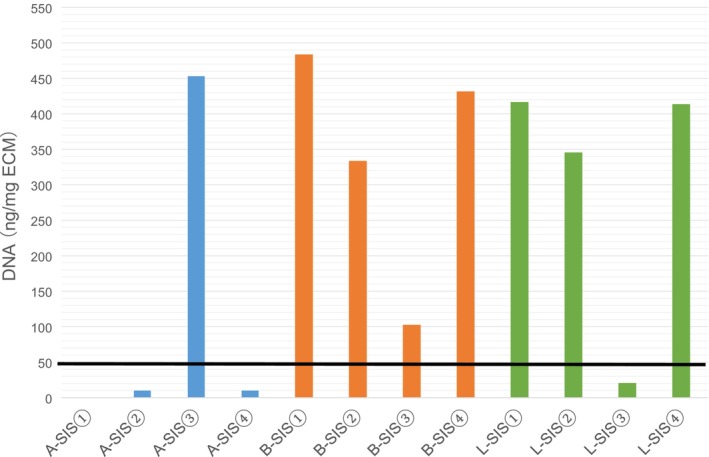
Remnant DNA contents. Remnant DNA (ng/mg of extracellular matrix dry weight) in small intestinal submucosal samples by A‐SIS, B‐SIS, and L‐SIS was quantified.

We examined four specimens per decellularization method with the residual amount of DNA for each SIS shown in Figure 2. Crapo et al. proposed a residual DNA level of 50 ng/mg of extracellular matrix dry weight or less as a criterion for decellularized tissue based on the Badylak method.^17^ In A‐SIS, the amount of residual DNA was 10 (0–453) ng/mg of extracellular matrix dry weight, with three out of four samples meeting the criterion. In B‐SIS, the amount of residual DNA was 382 (102–483) ng/mg of extracellular matrix dry weight; none of the four samples met the criterion. In L‐SIS, the amount of residual DNA was 379 (20–416) ng/mg of extracellular matrix dry weight, and only one out of four samples met the criterion.

### 3.3 Growth factor content of decellularized human SIS

**FIGURE 3:**
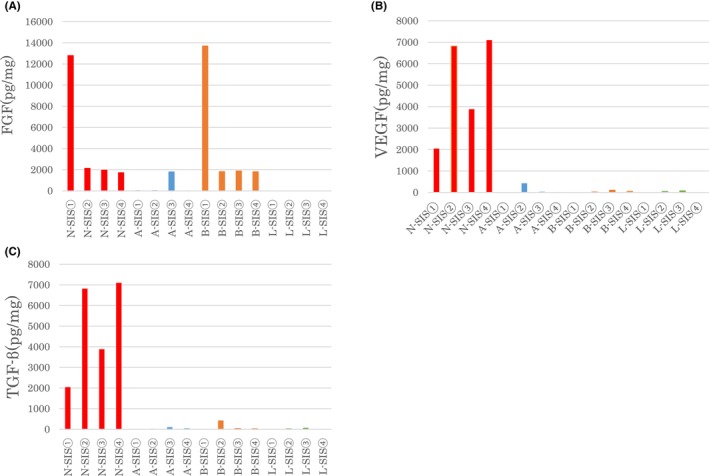
Residual growth factors in decellularized human small intestinal submucosa (SIS). Three types of growth factors, fibroblast growth factor (FGF), vascular endothelial growth factor (VEGF) and transforming growth factor β (TGF‐β), were measured by enzyme‐linked immunosorbent assay. (A) The concentration of FGF in each SIS. (B) The concentration of VEGF in each SIS. (C) The concentration of TGF‐β in each SIS.

Three growth factors (FGF, VEGF, TGF‐β) were measured in all samples by ELISA (Figure 3). The level of FGF was 2040 (1712–12,784) pg/mg in N‐SIS, 55 (52–1793) pg/mg in A‐SIS, 1850 (1806–13,691) pg/mg in B‐SIS, and 0 (0–0) pg/mg in L‐SIS. The level of VEGF was 5349 (2043–7102) pg/mg in N‐SIS, 26 (10–426) pg/mg in A‐SIS, 33 (1–102) pg/mg in B‐SIS, and 21 (0–71) pg/mg in L‐SIS. The level of TGF‐β was 65 (20–207) pg/mg in N‐SIS, 3 (0–160) pg/mg in A‐SIS, 0 (0–17) pg/mg in B‐SIS, and 0 (0–8) pg/mg in L‐SIS.

### 3.4 Cell proliferation and survival by co‐culture of decellularized human SIS and mouse BMSCs

**FIGURE 4:**
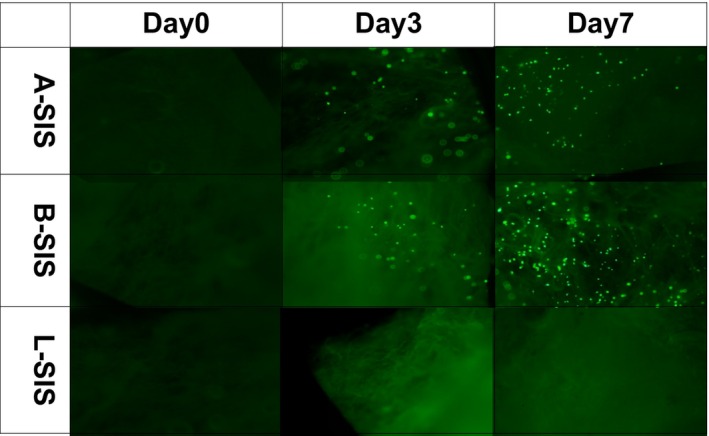
Representative images of co‐cultured mouse bone marrow‐derived mesenchymal stem cells (BMSCs) and decellularized human small intestinal submucosa on days 0, 3, and 7. Viable cells were stained with Cellstain® Calcein‐AM solution (Fujifilm, Tokyo, Japan) and observed under a fluorescent microscope BMSC engraftment increased over time in A‐SIS and B‐SIS, whereas no BMSC engraftment was observed in L‐SIS.

The representative cell proliferation and survival demonstrated upon co‐culture of mouse BMSCs and decellularized SIS are shown in Figure 4. A‐SIS and B‐SIS derived samples showed good proliferative activity and viable cell numbers, which increased over time. Conversely, L‐SIS decellularized tissue did not show viable cell engraftment.

**FIGURE 5:**
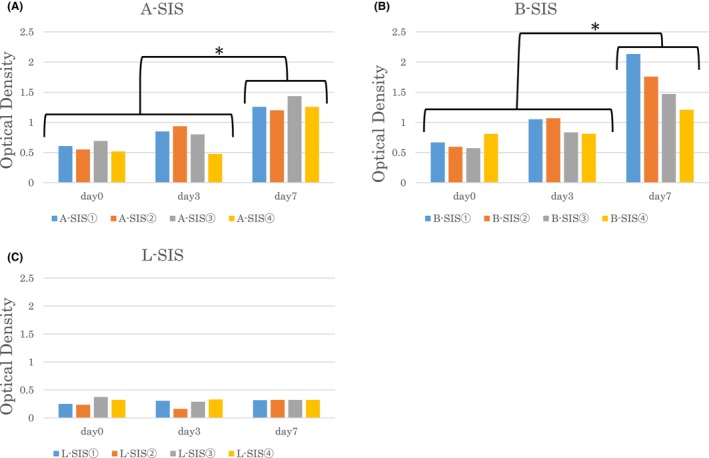
The optical density of viable cells upon co‐culture of mouse bone marrow‐derived mesenchymal stem cells (BMSCs) and decellularized human small intestinal submucosa (SIS) on days 0, 3, and 7. The optical density of viable cells was measured by Premix WST‐1 Cell Proliferation Assay System™ (Takara Bio, Kusatsu, Japan). In A‐SIS and B‐SIS, the number of cells increased continuously over time. The asterisk (*) represents significant differences in optical density values. Luo's method (L‐SIS) did not show an increase in cells.

The optical density and viable cell growth rates of each decellularized SIS are shown in Figure 5. In A‐SIS, proliferation rates increased over time: 0.58 (0.52–0.69) on day 0, 0.82 (0.48–0.94) on day 3, and 1.35 (1.26–1.43) on day 7. The proliferation rate at day 7 was also significantly higher than those on days 0 and 3 (*p* = .04). In B‐SIS, proliferation rates also increased over time: 0.63 (0.57–0.81) on day 0, 0.94 (0.81–1.07) on day 3, and 1.61 (1.21–2.13) on day 7. The proliferation rate at day 7 was significantly higher than those on days 0 and 3 (*p* = .03). Cells were not viable using the L‐SIS method. Proliferation rates failed to increase over time: 0.31 (0.23–0.62) on day 0, 0.31 (0.16–0.33) on day 3, and 0.32 (0.31–0.32) on day 7. Cell proliferation rates in A‐SIS and B‐SIS were statistically significant when compared with that of L‐SIS on day 7 (A‐SIS vs. L‐SIS: *p* = .04, B‐SIS vs. L‐SIS: *p* = .02). However, there was no significant difference in proliferation between B‐SIS and A‐SIS on day 7 (*p* = .35).

## 4 DISCUSSION

First, we decellularized porcine SIS by the method of Luo and obtained results equivalent to those reported by Luo et al.^9^ Based on this, we decellularized human SIS by Luo's method, but the results were different from porcine SIS. Therefore, in this study, human SIS was decellularized using three existing methods successfully optimized for porcine intestinal samples. Of all protocols tested, Abraham's method most adequately satisfies the criteria for decellularized tissue, with optimal survival and proliferation of cells demonstrated in co‐culture with mouse BMSCs.

Decellularization methods are roughly classified into three types, chemical, biological, and physical, with each method defined by its characteristic benefit profile. Interestingly, many current studies combine multiple methods to prepare decellularized tissue.^18^ Abraham's method involves a chemical method of prolonged incubation in acid/base (HCL/NaOH) and a final stage of physical agitation in sterile purified water.^8^ Badylak's method combines chemical incubation in peracetic acid with the physical method of continuous stirring.^7^ Luo's method decellularizes cells by using all three methods: stirring/agitation (physical), use of ethanol and peracetic acid (chemical), and incubation with digestive enzymes (biological).^9^ Yanhui et al. compared the characteristics of these three decellularization protocols in porcine SIS and reported that all methods met the criteria for decellularization.^12^ They further noted that Badylak's method had the lowest degree of damage to the extracellular matrix, while Luo's method had the greatest degree of damage.^12^

In our study, only Abraham's method met the criteria for decellularization of human SIS. The effectiveness of decellularization methods varies depending on the tissue type, thickness, density, and species of origin.^17^ Abraham's method required approximately 44 h for decellularization, while Badylak's method required approximately 3 h—a relatively short period of time. Although Luo's method uses chemical, biological, and physical aspects of decellularization, human SIS did not meet the criteria for decellularization. We theorize that this may be because of the shorter acid/base chemical incubation (30 min) in Luo's method compared with that (42 h) of Abraham's method. Therefore, it appears that chemical isolation is effective for decellularization of human SIS.

Regarding growth factors, Yanhui et al. reported that in porcine SIS, minimal damage to the extracellular matrix led to persistence of more growth factors.^12^ Similarly, in this study, Badylak's method had the most detectable growth factors and Luo's had the least. However, the quantity of growth factors was significantly reduced compared with N‐SIS.

Next, we observed the effects of persistent growth factors in decellularized human SIS on viability and proliferation of cells (mesenchymal stem cells). This experiment was performed in the study of Yanhui et al., and mouse bone marrow‐derived mesenchymal stem cells were used, which were also used in our study.^12^ Cells were non‐viable using L‐SIS but were able to proliferate using A‐SIS and B‐SIS despite low growth factor content. This suggests that A‐SIS and B‐SIS protocols led to residual growth factors sufficient for cell survival, despite levels being lower than those seen in N‐SIS.

When allografts are performed with human SIS, the criteria for decellularized tissue may be less stringent than those for xenografts. Therefore, in the decellularization of human SIS, Abraham's method, which causes less damage to the extracellular matrix and preserves more growth factors, is considered to be the most suitable method. In future, we intend to examine the original decellularization of human SIS based on Abraham's method.

There are several limitations to this study. Primarily, case numbers are low because small bowel specimens are not routinely obtained by informed consent from patients undergoing certain standard treatments. Next, in this study, we used three representative decellularization methods currently reported. However, there are many other methods besides these.^17^ In human‐derived tissue, we find Abraham's method most useful; however, other more efficient methods may exist. Furthermore, we confirmed engraftment and proliferation of viable cells even with low levels of growth factors. However, it is unclear how the three growth factors measured are involved in viable cell engraftment and proliferation, and which of the three is most important. Once the roles of these three growth factors are better understood, it is necessary to consider decellularization based on which growth factor should be retained in greatest concentrations. In conclusion, Abraham's method met the criteria for efficient decellularization and maintenance of cell viability and proliferation. It is considered most suitable for decellularization of human SIS although further work is required to optimize human intestinal scaffold generation.

## FUNDING INFORMATION

The authors received no funding for this article.

## CONFLICT OF INTEREST STATEMENT

The authors have no conflicts of interest to declare.
